# Case report: Clinical management of freshwater stingray wounds using negative pressure therapy

**DOI:** 10.3389/fmed.2025.1536540

**Published:** 2025-03-10

**Authors:** Janio J. M. Nattrodt, Victória A. Bezerra-de-Freitas, Ana Paula S. S. Merval, Eloise T. Filardi, Felipe A. Cerni, Domingos S. M. Dantas, Alysson B. M. Lins, Luis E. B. Galan, Roberto C. Carbonell, Manuela B. Pucca

**Affiliations:** ^1^General Hospital of Roraima, Boa Vista, Brazil; ^2^Medical School, Federal University of Roraima (UFRR), Boa Vista, Brazil; ^3^Graduate Program in Health and Science (PROCISA), Federal University of Roraima (UFRR), Boa Vista, Brazil; ^4^Graduate Program in Bioscience and Biotechnology Applied to Pharmacy, School of Pharmaceutical Sciences, São Paulo State University (UNESP), Araraquara, Brazil; ^5^Programa Doutoral de Bioética da Faculdade de Medicina do Porto, Porto, Portugal; ^6^Department of Clinical Analysis, School of Pharmaceutical Sciences, São Paulo State University (UNESP), Araraquara, Brazil

**Keywords:** ichthyism, stingray accident, stingray management, epidemiology, Amazon

## Abstract

Stingray injuries represent a significant occupational hazard, particularly for fishermen, and are commonly caused by freshwater stingrays of the Potamotrygonidae family. These stingrays are equipped with a sharp, bilaterally serrated spine that delivers venom, inducing vasoconstriction, severe pain, and ischemia. Such injuries are not only intensely painful but also debilitating, often rendering victims unable to work for weeks or even months. Traditional self-treatment practices, including the application of urine and herbal remedies, are widely relied upon in affected communities but are scientifically unproven and frequently lead to delayed or suboptimal care. This study presents two clinical cases of freshwater stingray envenomation from Roraima, the northernmost state of Brazil located within the Amazon Rainforest. Both cases were managed at the infectious disease unit of the General Hospital in Boa Vista, the state capital. Patients received evidence-based medical care, including intravenous antibiotic therapy and surgical debridement to remove necrotic and devitalized tissue. In one case, advanced negative pressure wound therapy (NPWT) was utilized during dressing changes, resulting in a clean wound devoid of edema and necrotic tissue, demonstrating the technique’s effectiveness in promoting wound healing. By accelerating wound healing and mitigating complications such as infections and chronic wounds, NPWT significantly enhance patient outcomes. Furthermore, this study underscores the limitations of traditional remedies and advocates for the adoption of evidence-based interventions, particularly in regions like the Brazilian Amazon, where access to healthcare can be challenging.

## Introduction

Brazil hosts the world’s largest river network, where venomous animals hold a significant ecological and public health presence. Among these, freshwater stingrays of the Potamotrygonidae family are particularly noteworthy. These generally docile creatures rarely exhibit aggression toward humans; however, they adopt defensive behaviors when accidentally stepped on or when their fins are inadvertently touched (1). In such cases, the stingray injects venom through its sharp, bilaterally serrated spine, resulting in irregular wounds or lacerations (2). Injuries caused by these animals are categorized under ichthyism. More specifically, acanthotoxic ichthyism, associated with venomous fish such as stingrays, is defined by traumatic or necrotic wounds accompanied by severe pain. The most commonly affected anatomical regions include the feet and heels in swimmers, whereas fishermen are particularly prone to hand injuries when handling stingrays (3, 4). The injuries present with a range of symptoms, including intense pain, skin necrosis, blister formation, ulceration, and fever. In severe cases, involving vital organ damage or bacterial infections, fatalities may occur (5). Victims often report pain disproportionate to the size of the injury, which typically subsides within 6 to 48 h but can persist for days or even weeks in some cases. The wounds usually exhibit jagged edges, profuse bleeding, and contamination with fragments of the stingray’s tegumentary layer (6).

Initial stages of envenomation are marked by erythema and edema around the wound, followed by central necrosis, which leads to tissue sloughing and the formation of deep ulcers. Systemic complications, such as nausea, vomiting, hypersalivation, sweating, respiratory depression, muscle fasciculations, and seizures, may also occur (7, 8). Epidemiological studies on freshwater stingray injuries in Brazil remain limited (1). While cases have been documented in the Paraná, Paraguay, and Araguaia river basins, the Amazon rainforest region accounts for the highest incidence of such accidents (9). Despite being a significant public health concern in the Amazon, these injuries receive comparatively less attention than snakebites and incidents involving venomous arthropods.

Given the lack of specific treatments for stingray injuries and the limited training healthcare professionals receive on this subject, disseminating information is crucial. Recommended first aid includes gently irrigating the wound with saline water to remove fragments of the spine, glandular tissue, and tegumentary debris. Spine removal should only be performed if the spine is superficially embedded and not penetrating vital areas like the neck, chest, or abdomen, or causing deep limb injuries. For significant bleeding, applying local pressure is essential (6).

Literature and clinical experience suggest immediate pain control through immersion of the affected limb in non-scalding hot water (approximately 60°C). Heat inactivates the polypeptide-based, thermolabile toxins—such as serotonin, phosphodiesterase, and 5-nucleotidase—that are responsible for pain and vasoconstriction. Early excision of necrotic tissue may be beneficial, although determining the extent of necrosis during early stages can be challenging. Chronic complications should be managed with wound healing protocols, and supportive care is recommended for systemic symptoms. Prophylactic tetanus vaccination, wound elevation, antibiotics, and surgical wound closure may also be required (1, 6).

Negative Pressure Wound Therapy (NPWT) is an effective approach for treating complex, traumatic, and surgical wounds, as well as burns, necrotic lesions, and pressure or diabetic ulcers. It has also been used for skin grafts to enhance vascularization and improve graft acceptance rates. NPWT has been successfully applied in various clinical scenarios, demonstrating its versatility and effectiveness. It has been used in complex surgical wounds, particularly dehiscent wounds, to improve healing by reducing the risk of infection. In diabetic foot ulcers, NPWT has been widely employed to aid healing and reduce the risk of amputations. It has also proven effective in managing traumatic wounds, accelerating recovery and enhancing the formation of healthy tissue. Additionally, NPWT has been utilized to stabilize and improve the integration of skin grafts, ensuring better vascularization and adherence. In burn cases, the therapy has helped reduce excess exudate, prevent infections, and create a favorable environment for recovery. These examples highlight the diverse applications of NPWT in clinical practice, consistently aiming to improve patient outcomes (10). Additionally, the combination of NPWT with dermal regeneration matrices was analyzed in a study published in the Brazilian Journal of Burns, which demonstrated the effectiveness of the technique in integrating skin grafts and accelerating the maturation time in patients with deep wounds and extensive burns (11). Moreover, a review demonstrated the application of NPWT in both acute and chronic wounds, emphasizing its ability to remove exudate and promote healing in severe infection cases (12). More recent studies further support these findings, demonstrating the applicability of NPWT in other clinical contexts. For instance, NPWT was used in preventing complications in surgical wounds following total knee arthroplasty, showing positive results such as reduced infections and faster wound closure (13). Those data confirm that NPWT is an effective and versatile tool in managing high-complexity wounds, offering safe and efficient solutions across different therapeutic settings.

Therefore, NPWT’s primary goals include cost-effectiveness, pain reduction, shorter hospital stays, and improved healing outcomes (14). The technique involves placing an open-pore hydrophobic polyurethane spongeor sterile gauze which comes into direct contact with the wound bed, covering the entire space, including cavities and tunnels. The sponge can be replaced within a time frame of 5 to 8 days depending on the clinical criteria. On top of this structure, a transparent and hermetic adhesive dressing is applied, isolating the area. A plastic tube connected to the dressing allows drainage of exudates into a reservoir linked to a computerized device, which continuously or intermittently regulates the negative pressure. The applied pressure ranges from −75 mmHg to −125 mmHg, depending on the complexity and needs of the wound, promoting more efficient healing. This process results in benefits such as the removal of fluids and inflammatory mediators, reduction of bacterial load, and approximation of the wound edges, facilitating closure (15).

The mechanisms of action of NPWT encompass both physical and biological aspects. Macrodeformation, promoted by negative pressure, reduces the wound size by bringing the edges closer together, while microdeformation acts at the cellular level, stimulating fibroblast proliferation and angiogenesis, which are crucial for tissue regeneration. Furthermore, the system creates a controlled moist environment, which accelerates the formation of granulation tissue and minimizes the risk of necrosis. Another significant benefit is the ability to remove exudates rich in bacteria and inflammatory substances, reducing the risk of infection and keeping the area cleaner and more prepared for natural healing processes. Studies highlight that NPWT is especially effective in traumatic wounds, postoperative wounds, pressure ulcers, and burns, showing shorter healing times and reduced complications compared to traditional techniques (11, 16, 17). NPWT is widely adopted globally to prepare wound beds for definitive closure (12). Given its therapeutic benefits, NPWT is a valuable option for managing stingray injuries.

The treatment of wounds caused by stingrays is particularly challenging due to the complexity of the injuries, which often involve deep trauma, bacterial infection, and venom contamination (18). Thus, NPWT promises to be a valuable approach for accelerating the healing of these wounds, offering benefits in both clinical recovery and improvement in patients’ life quality. By removing exudates, reducing inflammation, and minimizing bacterial load, NPWT creates an ideal environment for tissue regeneration and accelerates wound closure. This can reduce hospitalization time and the need for additional treatments, allowing patients to return to their daily activities more quickly, which directly impacts their psychological and emotional well-being. For patients with stingray injuries, especially in visible areas, faster healing can restore self-esteem and confidence, leading to a more peaceful physical and emotional recovery. This report presents two cases of fishermen injured by freshwater stingrays, both of whom developed severe ulcerative lesions accompanied by significant edema. These injuries necessitated specialized treatment, including the surgical debridement of necrotic and devitalized tissue to promote wound healing. The cases are compared based on the therapeutic approach: one utilizing negative pressure wound therapy, which effectively reduced edema and facilitated wound healing, and the other treated without this advanced method.

## Case series presentation

### Case report 1

A 59-year-old male fisherman resident of the municipality of Mucajaí, Roraima, with a medical history of hypertension and type 2 diabetes mellitus, under treatment with losartan, metformin, and glibenclamide, presented to the emergency department of the General Hospital of Roraima (HGR) on May 12, 2023. The patient sought medical attention 6 days after sustaining a stingray injury to the lateral malleolar region of his left ankle. He reported severe pain accompanied by significant localized edema and hyperemia. Prior to this visit, the patient had sought care at a local urgent care facility, where it was prescribed cephalexin, which he used for 3 days without improvement.

On May 14, 2023, he was transferred to the infectious disease service of the HGR. The laboratory tests revealed leukocytosis, neutrophilia, and elevated C-reactive protein (CRP) levels, consistent with a bacterial infection secondary to the stingray injury (Table 1). During hospitalization, the patient was treated with intravenous ciprofloxacin (400 mg every 12 h) and clindamycin (600 mg every 6 h) for 18 days. Additionally, his treatment included daily conventional wound care using collagenase and mechanical surgical debridement to manage the necrotic tissue and promote healing.

**Table 1:** Evolution of laboratory findings following freshwater stingray injury (Case 01)*.

Date	13/05/2023	16/05/2023	21/05/2023	30/05/2023
Leukocytes (x10^3^/μL)	13.18	9.7	7.41	5.7
Neutrophils (%)	76.4	62.2	53.9	57.3
Lymphocytes (%)	13.1	23.2	31.7	27.4
Hemoglobin (g/dL)	12	11.7	12	11.2
Hematocrit (%)	36.9	35.3	36.8	35.1
Platelets (x10^3^/μL)	283	355	475	451
Coagulation Tests	TAP 14 INR 1.07 TTPA 32.1	TAP 13.4 INR 1.01 TTPA 28	TP 14.3 INR 1.09 TTPA 29.1	TP 14.3 INR 1.2 TTPA 29.2
C-Reactive Protein (mg/L)	178.01	164.11	56.47	17.46
Albumin (g/dL)		2,93		
Urea/Creatinine (mg/dL)	26.76/1.07	21,52/0,98	31.5/ 1.02	29.83/1.14
AST/ALT (U/L)	12.26/15.5	11.95/11.39	12.14/ 14.71	13.46/ 15.12
Total Bilirubin / Direct Bilirubin / Indirect Bilirubin (mg/dL)	–	–	0.25/0.13/0.12	–
LDH (U/L)	–	–	–	240,19

*This table illustrates the progression of laboratory results during the hospitalization of Case 01.

**Figure 1:**
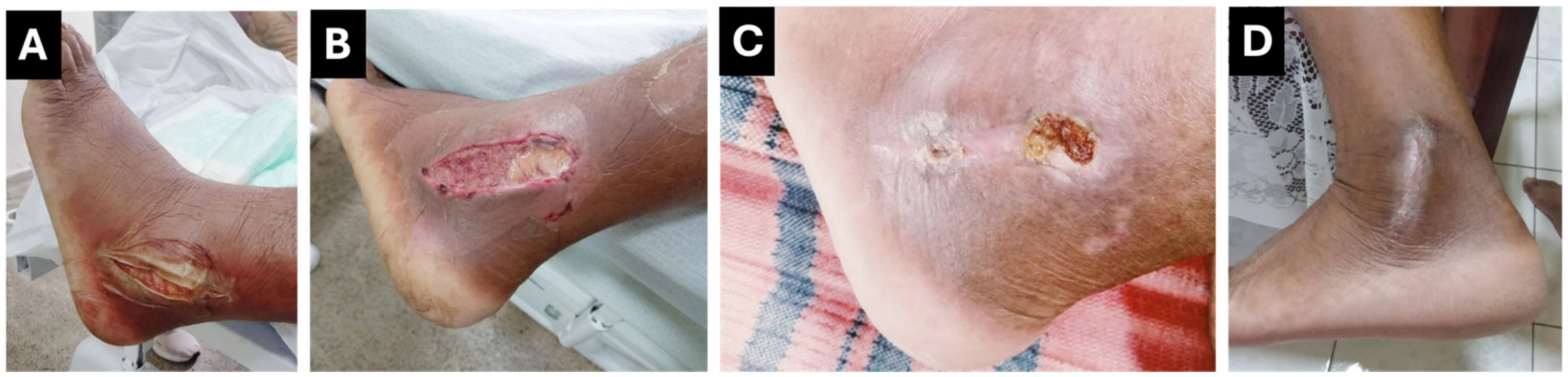
Case 1: Progression of Wound Healing Following Freshwater Stingray Injury. **(A)** Ulcerated lesion with devitalized tissue and significant limb edema. **(B)** Post-operative wound after debridement and irrigation, with healthier edges and reduced edema. **(C)** Outpatient follow-up showing substantial healing. **(D**) Fully healed wound 6 months after hospital discharge.

The wound on the patient’s left ankle demonstrated a favorable progression, with effective infection control, the absence of necrotic tissue, and the development of healthy granulation tissue (Figure 1). These improvements were accompanied by significant normalization of laboratory findings. The patient was subsequently evaluated by the plastic surgery team, which recommended hospital discharge with continued outpatient follow-up at the complex wound care clinic. The post-discharge treatment plan included alternate-day wound dressings and regular monitoring for secondary intention healing, which was successfully achieved 6 months after discharge.

### Case report 2

A 29-year-old male fisherman from Amajari, Roraima, with no prior medical history and use of routine medications, was admitted to the General Hospital of Roraima (HGR) on May 6, 2023, 2 days after a stingray injury to the medial malleolar region, extending to the dorsum of the right foot. Upon admission, he presented with fever, edema, severe pain in the right lower limb, necrosis, and purulent discharge from the wound. Hospitalization was initiated, and intravenous antibiotic therapy with ceftriaxone and clindamycin was started. The patient was transferred to Block B under the care of the infectious disease team on May 8, 2023.

Laboratory tests (Table 2) revealed leukocytosis (30 ×10^3^/μL), neutrophilia (94.8%), and an elevated C-reactive protein (CRP) level of 159 mg/L. Based on these findings, antibiotic therapy was escalated to piperacillin-tazobactam (4.5 g IV every 6 h).

**Table 2:** Evolution of laboratory findings following freshwater stingray injury (Case 02)*.

Date	06/05/2023	11/05/2023	14/05/2023	16/05/2023	21/05/2023	30/05/2023
Leukocytes (x10^3^/μL)	30	19.26	14.09	8.37	6.42	4.61
Neutrophils (%)	94.8	83.6	75.5	71.5	57.7	45.7
Lymphocytes (%)	1.9	9.3	18.6	22.7	32.4	37.6
Hemoglobin (g/dL)	13.6	12.5	13	13	12.5	12.1
Hematocrit (%)	39.5	37.4	39	39.9	38	36.1
Platelets (x10^3^/μL)	209	301	400	411	487	278
Coagulation Tests	–	TAP13.1 INR 1 TTPA 35.7	TAP 13.3 INR 1 TTPA 34.3	TAP 13.4 INR 1.01 TTPA 35	TP 14.4 RNI 1.1 TTPA 37.2	TP 13.3 RNI 1.11 TTPA 28.6
C-Reactive Protein (mg/L)	159.51	112.52	68.4	21.51	11.44	4.41
Albumin (g/dL)	–	2.69	–	3.21	3.5	–
Urea/Creatinine (mg/dL)	24.49/1.11	21.88/0.86	16.4/1.01	28.95/0.9	23.1/ 0.9	17.01/ 1.17
AST/ALT (U/L)	48.01/72.58	52.37/98.3	36.56/66.21	38.13/70.3	26.33/ 37.43	19.45/ 25.6
Total Bilirubin/Direct Bilirubin/Indirect Bilirubin (mg/dL)	–	–	0.54/0.21/0.33	–	0.43/0.25/0.18	0.37/0.23/0.14
LDH (U/L)	–	324.83	281.95	–	–	237.8

*This table illustrates the progression of laboratory results during the hospitalization of Case 02. In yellow values referring to the exams before the NPWT; in blue values referring during the NPWT; and in green values referring after the NPWT.

In yellow values referring to the exams before the NPWT; in blue values referring during the NPWT; and in green values referring after the NPWT.

**Figure 2:**
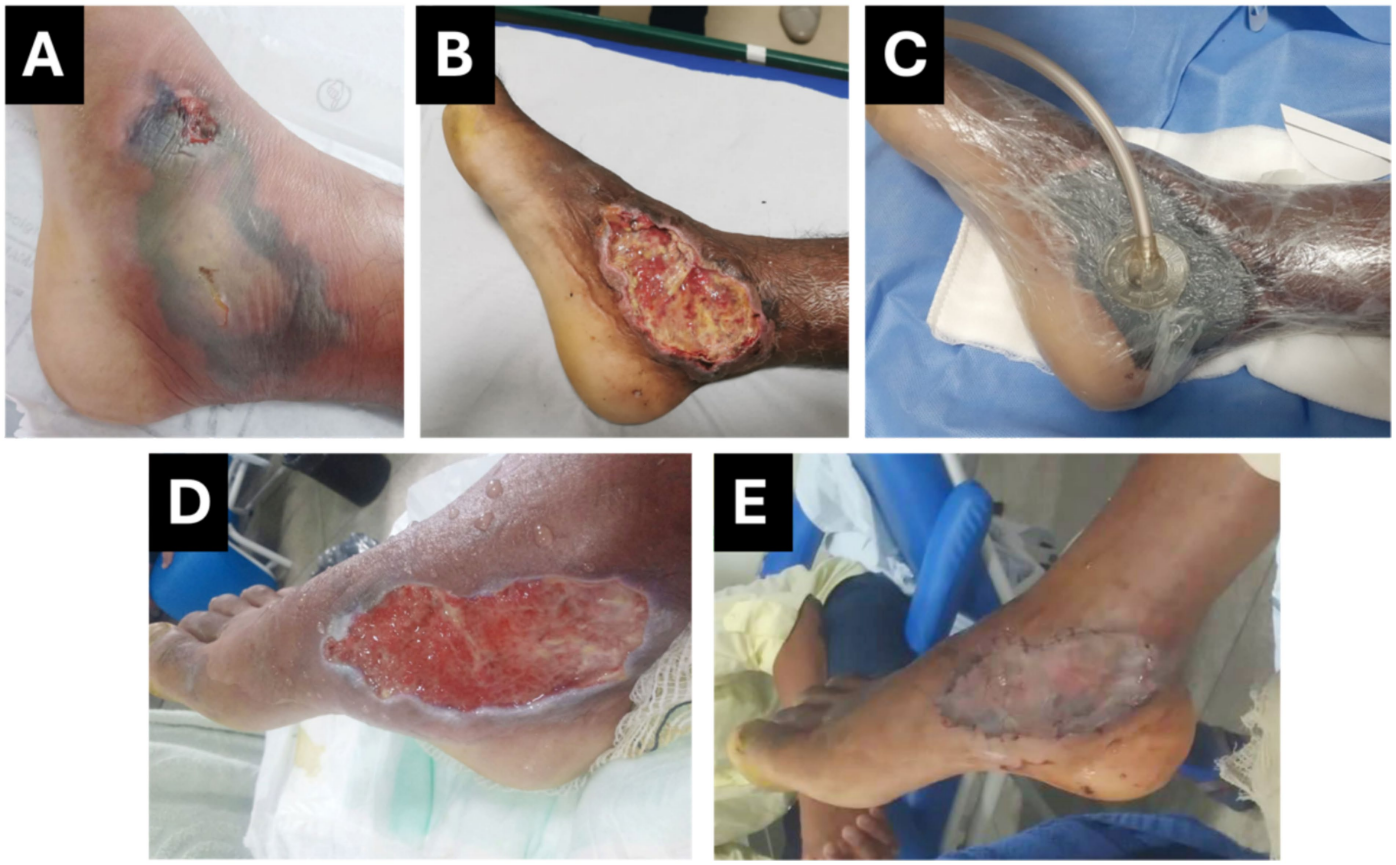
Case 2: Progression of Wound Healing Following Freshwater Stingray Injury. **(A)** Ulcerated lesion with devitalized, flaccid tissue, and extensive edema and necrosis. **(B)** Immediate post-operative appearance following surgical debridement of necrotic and devitalized tissue. **(C)** Application of Negative Pressure Wound Therapy (NPWT). **(D)** Wound after NPWT removal, showing granulation tissue, absence of purulent exudate, and no necrotic areas. **(E)** Immediate post-operative appearance after autologous skin grafting.

On May 17, surgical debridement was performed to remove necrotic tissue, and 48 h later (May 19), Negative Pressure Wound Therapy (NPWT) was initiated (Figure 2). Pressure was applied consistently and maintained at 125 mmHg during the therapy. Following favorable progress with NPWT, granulation tissue covered a significant portion of the wound, with only minor residual necrotic areas. The wound dressing was removed on May 27, after 8 days. A second surgical procedure was performed by the plastic surgery team, involving debridement of the remaining necrotic tissue and the application of an autologous skin graft on May 29.

The patient was discharged on June 6, 2023, with significant laboratory improvement. The skin graft showed good acceptance, and the patient was scheduled for outpatient follow-up to monitor the wound until complete resolution.

## Discussion

Stingray injuries pose a serious public health issue, especially in regions like the Amazon, where there is a high overlap between human activity in riverine areas and freshwater stingray habitats. Epidemiological data show that men, particularly fishermen, riverine workers, and individuals engaged in recreational activities in shallow waters, are most commonly affected. Injuries typically involve the feet and lower limbs and are more frequent during the dry season when river levels drop, increasing human exposure in these areas (19). Despite their prevalence, these injuries are often underreported and receive less attention in public health discussions compared to other envenomations such as snakebites and spider bites.

The complications from stingray injuries can be severe, including intense pain, significant edema, erythema, tissue necrosis, and the development of deep ulcers. These wounds often require surgical debridement to remove dead tissue, as well as antibiotic therapy to treat secondary bacterial infections (6). In addition to local complications, some patients may experience systemic symptoms. These range from mild issues such as nausea and vomiting to severe manifestations like respiratory depression, muscle fasciculations, and convulsions, which further complicate management (7, 8).

A study conducted in Belém, Pará, Brazil, examined stingray injuries over a 10-year period, revealing that nearly 89.2% of cases required wound debridement due to localized infections and necrosis (19). This highlights the necessity of timely medical care and effective wound management strategies. However, traditional healing by secondary intention, while sometimes effective, can be prolonged. This extended recovery increases healthcare costs, delays patients’ return to daily activities and diminishes overall quality of life.

Negative Pressure Wound Therapy (NPWT) has emerged as an innovative and effective approach to wound management in such cases. By applying controlled subatmospheric pressure, NPWT creates an optimal healing environment. It reduces edema, lowers bacterial counts, removes wound exudates and debris, and promotes the formation of granulation tissue. Additionally, NPWT enhances blood flow to the wound and minimizes the risk of secondary infections, making it an invaluable tool for managing complex injuries, including those caused by envenomation (20).

In this study, Case 01 followed a conventional treatment approach and experienced a longer recovery period, with wound closure achieved by secondary intention. Although the patient ultimately healed, the extended timeline required repeated wound care, which increased financial and emotional strain on both the patient and their family. Prolonged hospitalization and delayed recovery also increased the risk of secondary complications, underscoring the limitations of traditional wound management methods (21, 22).

Conversely, Case 02 illustrates the transformative potential of NPWT. After surgical debridement, NPWT helped establish a clean wound bed with healthy granulation tissue, free from purulent exudate, necrosis, or edema. This prepared the wound for early skin grafting and tertiary closure, leading to faster healing, shorter hospitalization, and improved cosmetic and functional outcomes. The reduced recovery time and fewer complications highlight the cost-effectiveness of NPWT, aligning with findings from previous studies that emphasize its benefits in complex wound care (23, 24).

The comparison between these cases demonstrates the clear advantages of NPWT over standard care. By promoting faster wound closure and reducing complications, NPWT offers a more efficient and patient-centered solution. In addition to its clinical benefits, NPWT also significantly contributes to improving the life quality by reducing the pain associated with stingray wounds, which is often intense due to the venom and infection. The decrease in edema and removal of inflammatory mediators provide noticeable relief, reducing the daily discomfort of patients and improving their activity levels (16, 17). For patients with deep injuries, the NPWT therapy can restore self-esteem by improving the appearance of the wound and reducing visible scarring.

Furthermore, its adaptability to various types of wounds, including those complicated by necrosis and infection, underscores its value in resource-constrained environments like the Amazon, where access to advanced medical treatments is often limited. Beyond individual patient benefits, NPWT has broader implications for public health. By reducing the length of hospital stays and minimizing complications, NPWT lowers overall healthcare costs, an essential consideration for regions with limited resources. Moreover, NPWT enables patients to feel more secure and less anxious and stressed about the recovery process, directly contributing to their emotional and psychological well-being. The therapy can also promote a more efficient physical recovery allowing individuals to re-engage in their daily routines with fewer limitations allowing the patients to typically return to their daily lives and work more quickly, lessening the socio-economic burden on families and communities (24–26).

However, the widespread implementation of NPWT faces challenges, particularly in remote and underserved areas. These include the availability of the necessary equipment, the need for trained professionals, and the financial cost of integrating NPWT into public healthcare systems. Addressing these barriers will require investments in infrastructure, training programs for healthcare providers, and strategic partnerships to make NPWT more accessible to those in need (27).

## Data Availability

The original contributions presented in the study are included in the article/supplementary material, further inquiries can be directed to the corresponding authors.

## References

[1] Garrone Neto D Haddad JV. Arraias em rios da região Sudeste do Brasil: locais de ocorrência e impactos sobre a população. Rev Soc Bras Med Trop. (2010) 43:82–8. doi: 10.1590/S0037-86822010000100018, PMID: 20305975

[2] Magalhaes KW Lima C Piran-Soares AA Marques EE Hiruma-Lima CA Lopes-Ferreira M. Biological and biochemical properties of the Brazilian Potamotrygon stingrays: *Potamotrygon* cf. *scobina* and *Potamotrygon* gr. *orbignyi*. Toxicon. (2006) 47:575–83. doi: 10.1016/j.toxicon.2006.01.028 16564065

[3] Lim YL Kumarasinghe SPW. Cutaneous injuries from marine animals. Singapore Med J. (2007) 48:e25–8. PMID: 17245501

[4] Blanc Brisset I Schaper A Pommier P De Haro L. Envenomation by Amazonian freshwater stingray *Potamotrygon motoro*: 2 cases reported in Europe. Toxicon. (2006) 47:32–4. doi: 10.1016/j.toxicon.2005.09.005, PMID: 16303158

[5] Lameiras JLV Duncan WLP. *Arraias de Água Doce (Chondrichthyes – Potamotrygonidae)*. Scientia Amazonia (2013).

[6] Ferroada de Arraia Lesões. *Intoxicação. Manuais MSD edição para profissionais*. Available at: https://www.msdmanuals.com/pt/profissional/lesões-intoxicação/mordidas-e-picadas/ferroada-de-arraia (Accessed January 21, 2025).

[7] Haddad V Neto DG De Paula Neto JB De Luna Marques FP Barbaro KC. Freshwater stingrays: study of epidemiologic, clinic and therapeutic aspects based on 84 envenomings in humans and some enzymatic activities of the venom. Toxicon. (2004) 43:287–94. doi: 10.1016/j.toxicon.2003.12.006, PMID: 15033327

[8] Forrester MB. Pattern of stingray injuries reported to Texas poison centers from 1998 to 2004. Hum Exp Toxicol. (2005) 24:639–42. doi: 10.1191/0960327105ht566oa, PMID: 16408617

[9] Charvet-Almeida P Góes ML. *Neotropical freshwater stingrays: Diversity and conservation status*. (2008). Available at: https://www.semanticscholar.org/paper/Neotropical-Freshwater-Stingrays%3A-diversity-and-Charvet-Almeida-G%C3%B3es/8bd95be25a3b3303aefbd13f014417ca40d1ac99 (Accessed January 21, 2025).

[10] Zaver V Kankanalu P. Negative pressure wound therapy. Treasure Island, FL: StatPearls Publishing (2025). 35015413

[11] Oliveira MES Soares FF Feijó R MJL P. Curativo de pressão negativa associado à matriz de regeneração dérmica: análise da pega e do tempo de maturação. Revista Brasileira de Queimaduras. (2014) 13:76–82.

[12] Dos Santos TL Da Silva AN De Sousa MB Costa MP Da Rocha JC. Terapia por pressão negativa no tratamento de feridas. Rev. Eletrôn. Acervo Saúde. (2019) 2019:e1231. doi: 10.25248/reas.e1231.2019

[13] Rosa AG Fortes RC Reis CMS Farias PVS. Efeitos da terapia por pressão negativa na prevenção de complicações em ferida cirúrgica de artoplastia total do joelho: um estudo baseado em evidências. OLEL. (2024) 22:e4490. doi: 10.55905/oelv22n5-025

[14] Borges MCM. *Terapia por pressão negativa no tratamento de lesões por pressão: revisão da literatura*. (2023). Available at: https://repositorio.pucgoias.edu.br/jspui/handle/123456789/5916 (Accessed January 21, 2025).

[15] Terapia de Feridas com Pressão Negativa. *Uma Revisão de Seu Uso em Trauma Ortopédico*. HARTMANN GROUP. Available at: https://www.hartmann.info/pt-br/blog-cientifico-hartmann/l/br/terapia-de-feridas-com-pressao-negativa-uma-revisao-de-seu-uso-em-trauma-ortopedico (Accessed January 21, 2025).

[16] De Souza BR Alencar ACDA Barletta CAB De Sousa AZSF Da Costa JB De Lima AB. Terapia por pressão negativa em feridas traumáticas. BJDV. (2021) 7:117100–13. doi: 10.34117/bjdv7n12-458

[17] Ferreira MC Paggiaro AO. Terapia por pressão negativa-vácuo. Rev Med (São Paulo). (2010) 89:142–6. doi: 10.11606/issn.1679-9836.v89i3/4p142-146

[18] Meyer PK. Stingray injuries. Wilderness Environ Med. (1997) 8:24–8. doi: 10.1580/1080-6032(1997)008[0024:si]2.3.co;2, PMID: 11990133

[19] De Oliveira P Parda P Pires WM Borba P Ferreira H Messias Oliveira Brito R. Freshwater stingray injuries in Belém, state of Pará, Brazil. J Health NPEPS. (2020) 5:99–115. doi: 10.30681/252610104423, PMID: 30304272

[20] Normandin S Safran T Winocour S Chu CK Vorstenbosch J Murphy AM. Negative pressure wound therapy: mechanism of action and clinical applications. Semin Plast Surg. (2021) 35:164–70. doi: 10.1055/s-0041-1731792, PMID: 34526864 PMC8432996

[21] McCaughan D Sheard L Cullum N Dumville J Chetter I. Patients’ perceptions and experiences of living with a surgical wound healing by secondary intention: a qualitative study. Int J Nurs Stud. (2018) 77:29–38. doi: 10.1016/j.ijnurstu.2017.09.015, PMID: 29031127 PMC5744862

[22] Chetter IC Oswald AV McGinnis E Stubbs N Arundel C Buckley H. Patients with surgical wounds healing by secondary intention: a prospective, cohort study. Int J Nurs Stud. (2019) 89:62–71. doi: 10.1016/j.ijnurstu.2018.09.011, PMID: 30343210

[23] Jones DDA Neves Filho WV Guimarães JDS Castro DDA Ferracini AM. The use of negative pressure wound therapy in the treatment of infected wounds. Case Stud Rev Bras Ortop (Engl Ed). (2016) 51:646–51. doi: 10.1016/j.rboe.2016.10.014, PMID: 28050534 PMC5198081

[24] Norman G Shi C Goh EL Murphy EM Reid A Chiverton L. Negative pressure wound therapy for surgical wounds healing by primary closure. Cochrane Database Syst Rev. (2022) 2022:CD009261. doi: 10.1002/14651858.CD009261.pub7, PMID: 35471497 PMC9040710

[25] Lima RVKS Coltro PS Farina Júnior JA. Negative pressure therapy for the treatment of complex wounds. Rev Col Bras Cir. (2017) 44:81–93. doi: 10.1590/0100-69912017001001, PMID: 28489215

[26] Novak A Wasim J Palmer P. The evidence-based principles of negative pressure wound therapy in Trauma & Orthopedics. TOORTHJ. (2014) 8:168–77. doi: 10.2174/1874325001408010168, PMID: 25067971 PMC4110388

[27] Murray-Ramcharan M Feltes Escurra M Engdahl R Gattorno FL. Negative-pressure wound therapy for the Management of Complex Surgical Wounds in a minority population. Cureus. (2024) 16:e56726. doi: 10.7759/cureus.56726, PMID: 38646389 PMC11032736

